# Reversible Stress Cardiomyopathy Presenting as Acute Coronary Syndrome with Elevated Troponin in the Absence of Regional Wall Motion Abnormalities: A Forme Fruste of Stress Cardiomyopathy?

**DOI:** 10.1155/2014/796202

**Published:** 2014-05-26

**Authors:** Mahesh Anantha Narayanan, Vimalkumar Veerappan Kandasamy, Satish Chandraprakasam, Aryan Mooss

**Affiliations:** ^1^Department of Internal Medicine, Creighton University School of Medicine 601 N 30th Street No. 5800, Omaha, NE 68131, USA; ^2^The Cardiac Center of Creighton University, 3006 Webster Street, Omaha, NE 68131, USA

## Abstract

We present a case of reversible stress cardiomyopathy in a surgical patient, described here as a forme fruste due to its atypical features. It is important to recognize such unusual presentation of stress cardiomyopathy that mimics acute coronary syndrome. Stress cardiomyopathy commonly presents as acute coronary syndrome and is characterized by typical or atypical variants of regional wall motion abnormalities. We report a 60-year-old Caucasian male with reversible stress cardiomyopathy following a sternal fracture fixation. Although the patient had several typical features of stress cardiomyopathy including physical stress, ST-segment elevation, elevated cardiac biomarkers and normal epicardial coronaries, there were few features that were atypical, including unusual age, gender, absence of regional wall motion abnormalities, high lateral ST elevation, and high troponin-ejection fraction product. In conclusion, this could represent a forme fruste of stress cardiomyopathy.

## 1. Introduction

Stress cardiomyopathy is a well-established entity and commonly presents as acute coronary syndrome (ACS). The condition, also known as apical ballooning syndrome, was first reported in Japan in the early 90s. It commonly affects elderly females and accounts for 1.7 to 2.2% of those presenting with symptoms of ACS. We report a forme fruste of stress cardiomyopathy without regional wall motion abnormality and high troponin-ejection fraction product.

## 2. Case Report

**Figure 1:**
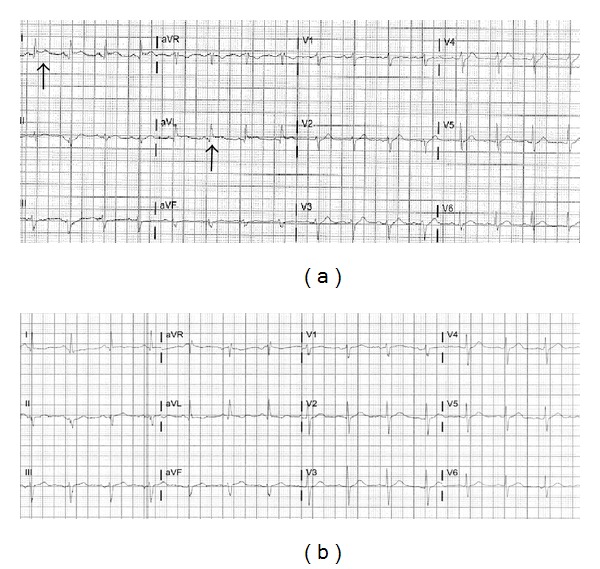
(a) EKG during chest pain-ST elevation in leads I and aVL. (b) Normal EKG after two days.

**Figure 2:**
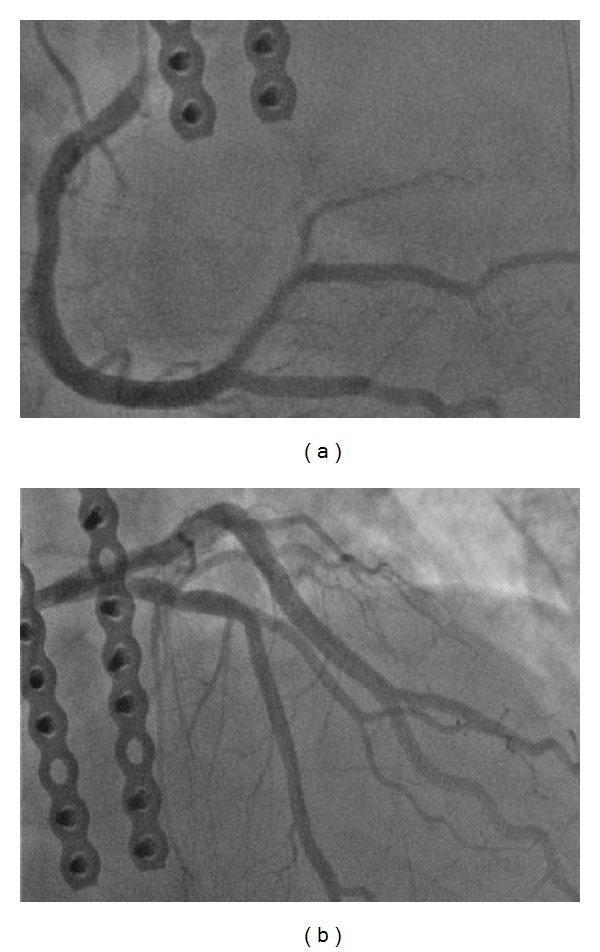
(a) Coronary angiogram showing right coronary artery. (b) Coronary angiogram showing left coronary artery.

A 60-year-old Caucasian male with a past history of heart failure with preserved ejection fraction, hypertension, deep venous thrombosis, pulmonary embolism (PE), inferior vena cava filter placement, long-term anticoagulation, and obstructive sleep apnea was admitted following a traumatic sternal fracture sustained 6 months ago that resulted in a malunion. He underwent an elective sternal fixation. Preoperative electrocardiogram (EKG) was within normal limits. Laboratory studies including complete blood count and chemistries were within normal limits. Postoperative course was complicated by incision site hematoma on postoperative day 6, requiring aspiration. On the following day, patient developed sudden onset of crushing, severe, substernal pain radiating to left arm and neck. Electrocardiogram (EKG) revealed ST-segment elevation of 1.5 mm in leads I and aVL (Figure 1(a)). Peak troponin I (Tn I) was 26 ng/mL (2600 ng/dL). Emergent coronary angiogram showed normal epicardial coronaries (Figures 2(a) and 2(b)). Left ventricular end diastolic pressure (LVEDP) was elevated at 36 mm Hg.  LV angiogram was deferred because of elevated LVEDP. Contrast echocardiogram showed ejection fraction of 60% with no identifiable regional wall motion abnormalities. Additional workup did not reveal any evidence of inflammation, infection, or acute PE. He improved with medical therapy on beta blockers. During followup, Tn I levels declined steadily and EKG changes normalized over the next two days (Figure 1(b)). The patient was discharged on beta blockers and appeared to be stable during followup.

## 3. Discussion

Stress cardiomyopathy, also known as reversible LV dysfunction, broken heart syndrome, or ampulla cardiomyopathy, is characterized by transient systolic dysfunction in the absence of obstructive coronary artery disease [1]. It is commonly precipitated by mental or physical stress, head trauma, acute illness, pheochromocytoma, subarachnoid hemorrhage, ischemic stroke, or exogenous catecholamine administration [2]. It is characterized by typical (apical ballooning) or atypical (midventricular or basal hypokinesis) variants of regional wall motion abnormalities [3]. In addition, transient EKG changes, modest troponin elevation, and regional wall motion abnormalities (RWMA) are noted with complete recovery in a few days to few weeks. Excessive catecholamine stimulation, metabolic disturbances, and microcirculatory dysfunction are proposed mechanisms [4]. Mayo Clinic criteria, proposed in 2004 and later modified in 2008, include (a) transient hypokinesis, akinesis, or dyskinesis in the LV mid-segments with or without apical involvement; RWMA extending beyond a single epicardial vascular distribution; the presence (often, but not always) of a stress trigger; (b) the absence of obstructive coronary disease or angiographic evidence of acute plaque rupture; (c) new electrocardiographic abnormalities (ST-segment elevation and/or T-wave inversion) or modest elevation of cardiac troponin levels in the serum; (d) the absence of pheochromocytoma or myocarditis. All four criteria must be met for diagnosis [5]. Myocardial perfusion imaging demonstrates reversible perfusion defects. Cardiac magnetic resonance imaging demonstrates RWMA, focal myocardial edema, and absence of late gadolinium enhancement [6].

Our patient's presentation was compatible with stress cardiomyopathy diagnostic criteria. This includes the presence of a stressful situation (fracture fixation), the typical clinical features including chest pain and dyspnea, ST-segment elevation in EKG, and troponin elevation with normal epicardial coronaries. But he had quite a few atypical features that did not fit in the diagnostic criteria. Our patient was a middle-aged male, whereas stress cardiomyopathy is commonly diagnosed in elderly females. The peak troponin in stress cardiomyopathy is usually less when compared to that in patients with STEMI. In addition, troponin-ejection fraction product (TEFP) obtained by multiplying peak troponin by ejection fraction obtained by echocardiography in our case was 156,000 and it does not fall in the range for stress cardiomyopathy. TEFP value ≥250 had a sensitivity of 95%, a specificity of 87%, a negative predictive value of 94%, a positive predictive value of 88%, and an overall accuracy of 91% to differentiate ST-segment elevation myocardial infarction from stress cardiomyopathy [7]. His troponin was disproportionately elevated in the range of an ST elevation MI. Also, in a typical case of stress cardiomyopathy, the ST-segment elevation involves the precordial leads, whereas our patient had a high lateral ST-segment elevation. He had preserved ejection fraction with no regional wall motion abnormalities on the echocardiogram, whereas typical stress cardiomyopathy will have wall motion abnormalities including apical ballooning. In our case, there was no other identifiable cause for his troponin elevation and EKG abnormality such as myocarditis, pericarditis, and PE. We suggest that this represents a forme fruste of stress cardiomyopathy. Our notion is supported by From et al. who suggested that abnormal stress echocardiograms with normal coronaries could represent a forme fruste of stress cardiomyopathy [8]. But to our knowledge, this is the first case report of stress cardiomyopathy without obvious regional wall motion abnormalities.
